# Hinokitiol Inhibits Melanogenesis via AKT/mTOR Signaling in B16F10 Mouse Melanoma Cells 

**DOI:** 10.3390/ijms17020248

**Published:** 2016-02-18

**Authors:** Yu-Tzu Tsao, Yu-Fen Huang, Chun-Yu Kuo, Yu-Chiang Lin, Wei-Cheng Chiang, Wei-Kuang Wang, Chia-Wei Hsu, Che-Hsin Lee

**Affiliations:** 1 Division of Nephrology, Department of Medicine, Taoyuan General Hospital, Taoyuan 330, Taiwan; tsaoyutzu@gmail.com; 2 Institute of Clinical Medicine, National Yang-Ming University, Taipei 11221, Taiwan; 3 Department of Microbiology, School of Medicine, China Medical University, Taichung 404, Taiwan; u103000425@cmu.edu.tw; 4 Graduate Institute of Basic Medical Science, School of Medicine, China Medical University, Taichung 404, Taiwan; u104082801@cmu.edu.tw (C.-Y.K.); s225701048@gmail.com (Y.-C.L.); m5210115@gmail.com (C.-W.H.); 5 Department of Environmental Engineering and Science, Feng Chia University, Taichung 40407, Taiwan; winton05@hotmail.com (W.-C.C.); wkwang@fcu.edu.tw (W.-K.W.); 6 Department of Biological Sciences, National Sun Yat-sen University, Kaohsiung 804, Taiwan

**Keywords:** hinokitiol, melanogenesis, autophagy, melanoma

## Abstract

H inokitiol purified from the heartwood of cupressaceous plants has had various biological functions of cell differentiation and growth. Hinokitiol has been demonstrated as having an important role in anti-inflammation and anti-bacteria effect, suggesting that it is potentially useful in therapies for hyperpigmentation. Previously, hinokitiol inhibited the production of melanin by inhibiting tyrosinase activity. The autophagic signaling pathway can induce hypopigmentation. This study is warranted to investigate the mechanism of hinokitiol-induced hypopigmentation through autophagy in B16F10 melanoma cells. The melanin contents and expression of microthphalmia associated transcription factor (MITF) and tyrosinase were inhibited by treatment with hinokitiol. Moreover, the phosphorylation of the protein express levels of phospho-protein kinase B (P-AKT) and phospho-mammalian targets of rapamycin (P-mTOR) were reduced after hinokitiol treatment. In addition, the microtubule associated protein 1 light chain 3 (LC3) -II and beclin 1 (autophagic markers) were increased after the B16F10 cell was treated with hinokitiol. Meanwhile, hinokitiol decreased cellular melanin contents in a dose-dependent manner. These findings establish that hinokitiol inhibited melanogenesis through the AKT/mTOR signaling pathway.

## 1. Introduction

**Figure 1:**
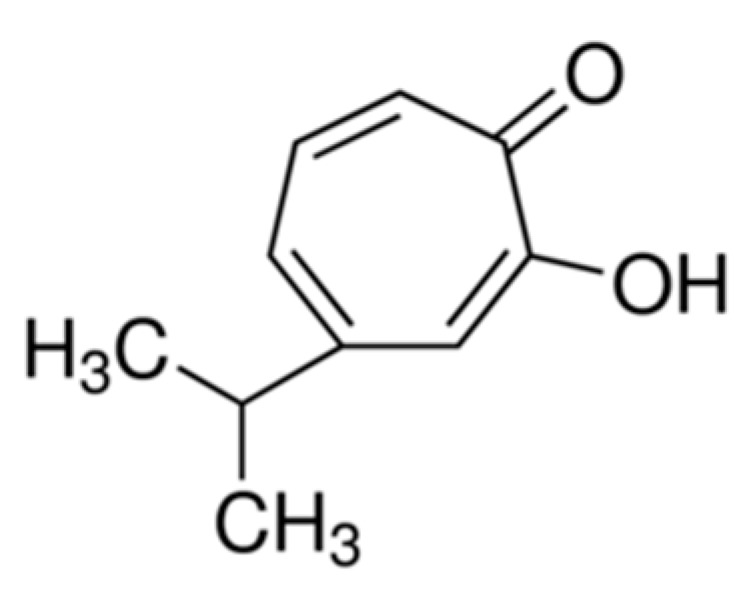
Chemical structure of hinokitiol.

Melanogenesis can be associated with number of diseases. Abnormal melanogenesis is involved in some human skin diseases, including melanoma and pigmentation disorders. Tyrosinase-related protein 1 (TRP1), and tyrosinase-related protein 2 (TRP2), and microphthalmia-associated transcription factor (MITF) are required for melanin synthesis [1]. Thus, some tyrosinase inhibitors including resveratrol and hinokitiol have been used in cosmetic applications [2,3,4,5]. Recently, some studies have demonstrated that autophagy may be involved in the regulation of melanogenesis [5,6,7]. Previously, we found that hinokitiol inhibited tumor growth by inducing autophagic signaling [8]. Hinokitiol is a tropolone-related compound purified from the heartwood of cupressaceous plants and has been used in hair tonics, cosmetics, and food as an antimicrobial agent (Figure 1) [8]. Although hinokitiol mainly inhibits the tyrosinase activity, the effect of hinokitiol on melanogenesis as an autophagy inducer has not been examined. Herein, hinokitiol was used as an autophagy regulator of hyperpigmentation.

## 2. Results

### 2.1. Anti-Melanogenic Activity of Hinokitiol in Vitro

**Figure 2:**
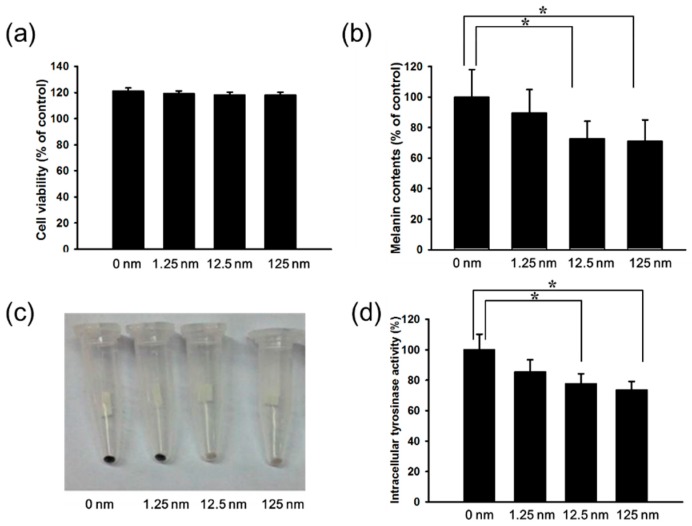
Effects of hinokitiol on cell viability and melanin production in B16F10 cell. B16F10 cells were treated with indicated concentrations of hinokitiol for 48 h. (**a**) Cell viability was measured by WST-1 assay; (**b**) Effect of hinokitiol on cellular melanin content; (**c**) Photograph of precipitated B16F10 melanoma cells. Cells were incubated for 48 h with (1.25–125 nM) and without hinokitiol; (**d**) Tyrosinase activity was measured. * *p* < 0.05 (mean ± SD, *n* = 6). Each experiment was repeated three times with similar results.

Herein, mouse B16F10 melanoma cells were used to investigate the anti-melanogenic activity of hinokitiol. The cell survival, and melanin content after B16F10 cells treated with hinokitiol are shown in Figure 1. We found the dose (1.25–125 nM) without significant cytotoxicity and used to evaluate the effects of hinokitiol on melanin production (Figure 2a). The production of melanin content in B16F10 cells was significant reduced in B16F10 cells after the treatment of hinokitiol. (Figure 2b,c). The increased treatment of hinokitiol significantly downregulated the tyrosinase activity in B16F10 cells (Figure 2d). These results demonstrated that hinokitiol inhibited the melanogenesis in B16F10 cells. 

### 2.2. Hinokitiol Reduced Tyrosinase and MITF Expression through the Phosphorylation-Protein Kinase B (P-AKT)/Phosphorylation-Mammalian Target of the Rapamycin (P-mTOR) Pathway

**Figure 3:**
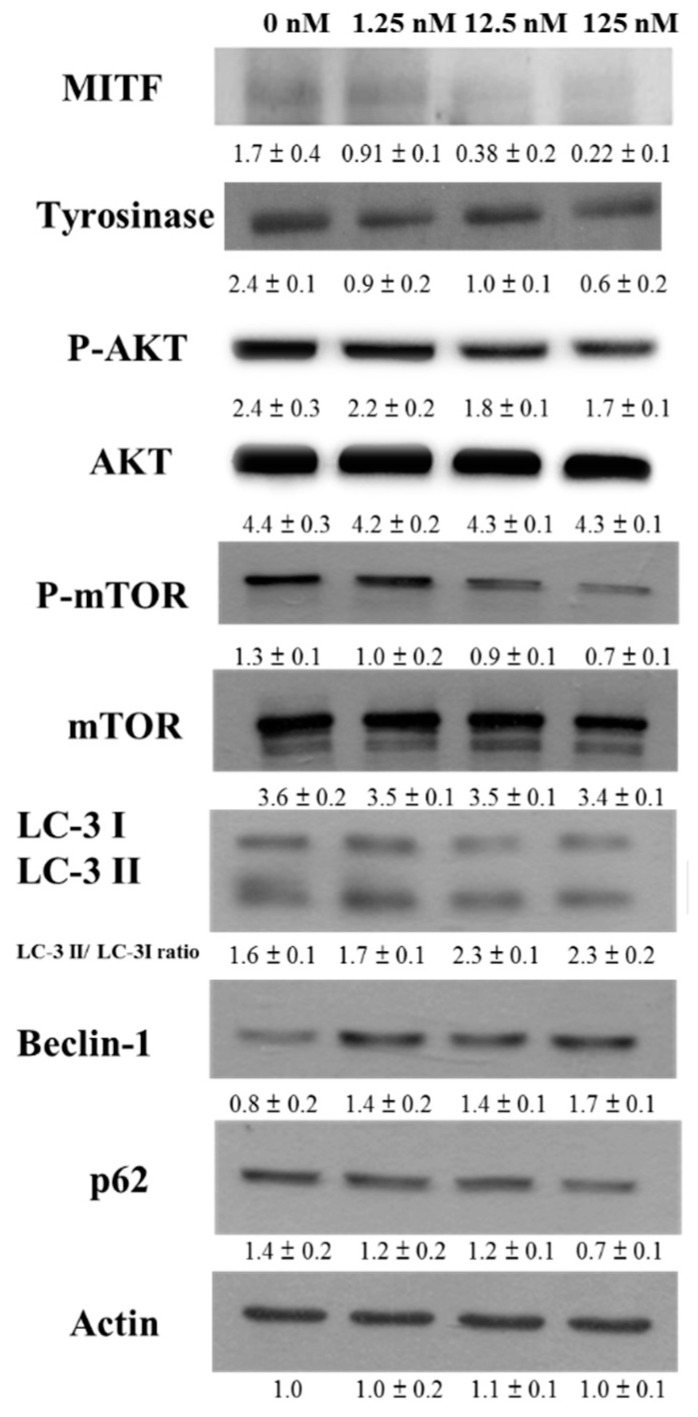
The expression levels of tyrosinase and microphthalmia-associated transcription factor (MITF) after hinokitiol treatment. B16F10 cells were treated with hinokitiol at the concentration of 1.25, 12.5 or 125 nM for 48 h. The protein expression was determined by immunoblotting. Inserted values indicated relative proteins expression in comparison with β-actin. Each experiment was repeated three times with similar results. (mean ± SD, *n* = 3).

To evaluate the mechanisms of the anti-melanogenic effects of hinokitiol, the expressions of tyrosinase were examined by Western blotting.Tyrosinase, a major enzyme, can induce melanogenesis. The expression levels of tyrosinase were decreased in hinokitiol-treated B16F10 cells (Figure 3). Because MITF can induce tyrosinase expression, the expression of MITF in B16F10 was measured. Hinokitiol significantly inhibited the expression of MITF in B16F10 cells. Because hinokitiol can influence the protein expression of MITF and reduce MITF-mediated transcriptional activity in B16F10 cells, we wanted to identify the signaling pathway affected by hinokitiol during melanogenesis. Some studies demonstrated that autophagy can reduce melanin synthesis [5,6,7]. Previous studies demonstrate that hinokitiol can induce autophagy through downregulating AKT/mTOR signal pathway [8]. In this study, the elevated phosphorylation of AKT, and mTOR in B16F10 cells was significantly diminished by hinokitiol treatment (Figure 3). The treatment with hinokitiol in B16F10 cells increased the conversion of microtubule associated protein 1 light chain 3 (LC3)-I to LC3-II. The autophagy can be affected by modulation of beclin 1 [9]. The expression of beclin1 was increased in B16F10 cells after the treatment of hinokitiol (Figure 3). p62 is itself degraded by autophagy [9]. As shown in Figure 3, the expression of p62 was decreased after treatment of hinokitiol in B16F10 cells. Taken together, treatment with hinokitiol decreased the phosphorylation of AKT and mTOR in a dose-dependent manner, indicating downregulation of the AKT/mTOR/MITF/tyrosinase pathway in B16F10 cells treated with hinokitiol. These results suggested that hinokitiol inhibited the expression of tyrosinase in B16F10 cells via reduction of the AKT/mTOR pathway.

### 2.3. Hinokitiol Reduced MITF via Inhibiting the Phosphorylation-AKT Signaling Pathway

**Figure 4:**
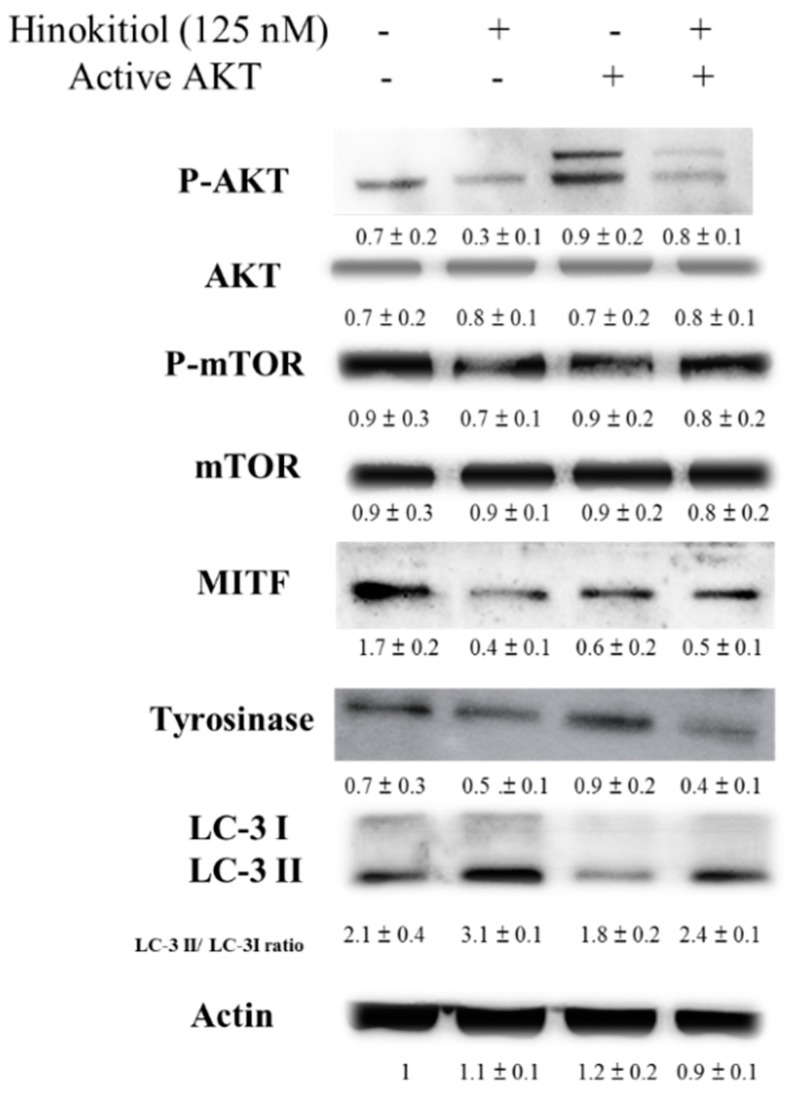
Constitutively active-AKT reduced the expression of tyrosinase and MITF. The B16F10 (10^5^) cells were transfected with constitutively active AKT plasmid (5 μg) for 16 h prior to treated with hinokitiol (125 nM) for 48 h. The expression of P-AKT, AKT, m-TOR, P-mTOR, MITF tyrosinase and LC3 protein in B16F10 cells was determined. The inserted values indicate relative protein expression compared to β-actin. This experiment was repeated with similar results. (mean ± SD, *n* = 3).

**Figure 5:**
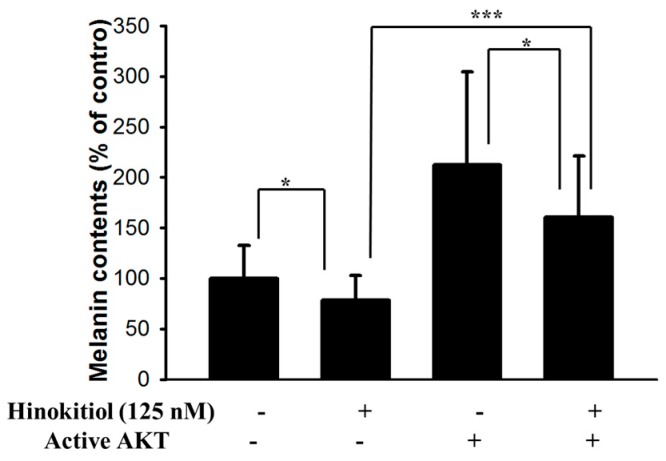
Hinokitiol reduces melanin content through AKT pathway. The B16F10 (10^5^) cells were transfected with constitutively active AKT plasmid (5 μg) for 16 h prior to treated with hinokitiol (125 nM) for 48 h. The melanin expression were measured. * *p* < 0.05; *** *p* < 0.001 (mean ± SD, *n* = 6). Each experiment was repeated three times with similar results.

Hinokitiol decreased MITF and tyrosinase expression in B16F10 cells through reducing AKT phosphorylation. The transfecting a plasmid bearing a constitutively active form of AKT can reversed the AKT/mTOR signaling pathway [10]. The transfecting a constitutively active form of AKT in B16F10 cells rescued the suppressive effect of hinokitiol on the AKT/mTOR signaling pathway (Figure 4). Transfection of a plasmid encoding constitutively active AKT increased the expression of MITF and tyrosinase by hinokitiol treatment. Hinokitiol decreased the melanin content of B16F10 cells (Figure 5). Transfection of a plasmid encoding constitutively active AKT increased the expression of melanin.The phenomenon was reversed after transfecting a plasmid encoding constitutively active AKT in B16F10 (Figure 5). The conversion of LC3-I to LC3-II by hinokitiol treatment in comparison with control transfection was reduced after transfecting constitutively active AKT plasmid. The markers of autophagy were also reversed. Our results indicated that inhibition of phosphorylation-AKT is necessary for the reduction of MITF and tyrosinase expression in B16F10 cells treated with hinokitiol.

## 3. Discussion

The synthesis of melanin is a multistep pathway in melanocytes. Melanogenesis is regulated through receptor-dependent and -independent pathways, in nutritional, hormonal redulation [11,12]. Melanin protects normal melanocytes and epidermis from various stresses [13]. The melanin can control epidermal homeostasis and can regulate the migration of melanoma [14]. The pigmentation disorders can affect functions and behavior not only of melanocytes but also surrounding cells in the skin [15,16]. Melanogenesis can affect behavior and clinical outcome of melanoma. The degradation of long-lived proteins and unwanted organelles in the cytosol is a cellular process through autophagy [17]. Autophagy pathway associates with anti-melanogenic agents in various of ways [5]. Previously, hinokitiol regulated the melanogenesis through modulating the activity of tyrosinase [2]. Autophagy can participate in melanosome degradation [7]. We found hinokitiol induced tumor cell death through autophagy in breast and colorectal cancers [8]. Herein, we want to identify the correlation of hinokitiol-induced autophagy and melanogenesis. Evidence shows that autophagy might participate in hyperpigmentation in melanocytes [6]. The AKT/mTOR pathway, one of the well-studied, was involved in autophagy signaling pathways [8,9]. Thus dose (125 nM) without cytotoxicity were observed and retained the activity for downregulation AKT/mTOR signaling. Hinokitiol could reduce the production of melanin. The MITF was reduced after treatment with hinokitol in a dose-dependent manner by Western blot analysis. In this study, we demonstrated that hinokitiol could inhibit the production of melanin by inhibition of AKT/mTOR signaling pathway.The AKT pathway is required for cell proliferation, cell survival and autophagy [6]. Inhibition of autophagy by transfecting constitutively-AKT plasmid suppressed hinokitiol-mediated anti-melanogenesis as well as autophagy. Some autophagy inducers, such as epigallocatehin gallate and resveratrol and 3’-hydroxydaidzein, reduced the production of melanin in melanocyte [5,7]. This study may not only investugate anti-melanogenic activity of hinokitiol, but also evaluate the signaling pathway of anti-melanogenic activities mediated by hinokitiol.

## 4. Experimental Section

### 4.1. Cells, Drugs and Plasmid

Murine B16F10 cells are cultured in Dulbecco’s modified Eagle’s medium (DMEM) supplemented with 50 μg/mL gentamicin, and 10% fetal bovine serum, at 37 °C in 5% CO_2_ [18]. Hinokitiol (99% purity), l-3,4-dihydroxyphenylalanine (l-DOPA), and dimethyl sulfoxide (DMSO) were purchased from Sigma-Aldrich (Sigma Aldrich, St. Louis, MO, USA). Dr. Chiau-Yuang Tsai provided the constitutively active AKT plasmid (Department of Molecular Immunology, Osaka University, Japan) [19].

### 4.2. Cell Proliferation Assay

B16F10 cells (10^5^/well) were treated with different concentration (1.25–125 nM) of hinokitiol in culture medium for 48 h. The colorimetric WST-1 assay was used for cell proliferation assay (Dojindo Labs, Tokyo, Japan) [20].

### 4.3. Evaluation of Melanin Content and Tyrosinase Activity 

After hinokitiol treatment, the cells were washed twice with PBS and harvested. The pellets of cells were solubilized in lysis buffer (20 mM sodium phosphate pH 6.8, 1% Triton X-100, 1 mM PMSF, and 1 mM EDTA). The melanin pellets were dissolved in Soluene-350 (Perkin-Elmer, Waltham, MA, USA) at 100 °C for 15 min after centrifugationa and the absorbance at 400 nm was measured [1,21]. To assay the tyrosinase activity, cell were dissolved in lysis buffer (1% SDS. 1% Tween 20, and 1 mM, l-DOPA) at 37 °C for 90 min and the absorbance was measured at 490 nm. The bicinchoninic acid (BCA) protein assay (Pierce Biotechnology, Rockford, IL, USA) was used for the protein content.

### 4.4. Western Blot Analysis

Proteins were fractionated on SDS-PAGE, and then transferred onto nitrocellulose membranes (Pall Life Science, Glen Cove, NY, USA), and probed with various antibodies against tyrosinase (Santa Cruz Biotechnology, Santa Cruz, CA, USA), MITF (Santa Cruz Biotechnology, Santa Cruz, CA, USA), beclin 1(Novus Biologicals, Littleton, CO, USA), LC3-I, LC3-II (Novus Biologicals, Littleton, CO, USA), p62 (Novus Biologicals, Littleton, CO, USA), mTOR (Cell Signaling, Danvers, MA, USA), phosphorylation-mTOR (Cell Signaling, Danvers, MA, USA), AKT (Santa Cruz Biotechnology, Santa Cruz, CA, USA), phosphorylation-AKT (Santa Cruz Biotechnology, Santa Cruz, CA, USA) or β-actin (Sigma-Aldrich, St. Louis, MO, USA). Horseradish peroxidase-conjugated anti-goat IgG, anti-mouse IgG or anti-rabbit IgG (Jackson, West Grove, PA, USA) was used as the secondary antibody, The signals were visualized with an enhanced chemiluminescence system (T-Pro Biotechnology, New Taipei City, Taiwan). The ImageJ software was used to quantified the signals (rsbweb.nih.gov/ij/) [22,23].

### 4.5. Statistical Analysis

The significant differences between groups were determined by unpaired, two-tailed Student’s *t* test. Any *p* value less than 0.05 is considered statistically significant. All data were expressed as mean ± standard deviation (SD).

## 5. Conclusions

In conclusion, hinokitiol as an anti-melanogenic agent with the potential to reduce hyperpigmentation. Furthermore, hinokitiol exerts downregulatory role in melanogenic signaling through AKT/mTOR signaling.
